# Structuring the psychological “State” assessment process for sports psychologists: a proposal for a theory-based holistic model

**DOI:** 10.3389/fpsyg.2026.1919803

**Published:** 2026-09-21

**Authors:** Yunus Emre Büyükbasmacı, Ünsal Tazegül

**Affiliations:** 1 Department of Recreation Management, Faculty of Applied Sciences, Karamanoglu Mehmetbey University, Karaman, Türkiye; 2 Department of Physical Education and Sports Teaching, Faculty of Sport Sciences, Igdir University, Igdir, Türkiye

**Keywords:** athletes, exercise and sport psychology, psychological assessment, sport psychologists, theoretical model

## Abstract

The aim of this perspective paper is to propose a theory-based holistic assessment model that structures the psychological state assessment process conducted by sport psychologists. It is observed that existing assessment approaches in the field of sports psychology largely focus on individual psychological variables, and measurement tools are mostly used independently of one another. This situation makes it difficult to comprehensively assess the multidimensional structure of the athlete's psychological profile and leads to a lack of standardization in application processes. The model was developed through a theory-driven process involving literature synthesis, conceptual framework development, expert review, and iterative refinement. The proposed model consists of a total of 16 psychological domains and 48 items, including motivation, concentration, anger, stress, anxiety, prosocial and antisocial behaviors, burnout, communication, mental resilience, perfectionism, sociotropy, autonomy, self-esteem, narcissism, self-confidence, and emotional intelligence. Each psychological domain is represented by three structured assessment items designed for practical use by sport psychologists. The conceptual framework and assessment items were reviewed by experts in sport psychology to enhance theoretical coherence and content relevance. It is expected that the model will enhance consistency in practice by systematizing the assessment processes of sport psychologists, provide opportunities for multidimensional analysis, support intervention planning, and contribute to filling the conceptual gap in the field.

## Introduction

Sports psychology has evolved into an interdisciplinary field that highlights the decisive role of psychological processes in athletic success, demonstrating that performance cannot be explained solely by physical and technical factors. As athletes and coaches strive for peak performance, they have also become increasingly aware of psychological factors, a trend that has made sports psychology an increasingly effective field (Türkay and Demir, 2021). Today, it is widely accepted that psychological variables such as motivation, attention, emotional regulation, self-confidence, and interpersonal interaction are of critical importance for the sustainability and consistency of performance (Weinberg and Gould, 2019). In this context, the systematic, reliable, and multidimensional assessment of athletes' psychological states has become a fundamental

necessity for both performance optimization and the promotion of psychological wellbeing. However, a review of the literature reveals that the assessment approaches used in sports psychology largely remain at the level of measurement tools. The development of a theory-based holistic assessment model that enables sport psychologists to assess athletes' psychological state in real time through a multidimensional, systematic, and practice-oriented approach is considered a critical necessity for improving the effectiveness, consistency, and quality of psychological assessment processes. In current practices, scales that typically focus on specific psychological variables are used; while these scales offer high-level psychometric properties, they fall short of addressing the multidimensional nature of athlete psychology within a comprehensive assessment process (DeVellis, 2017; Worthington and Whittaker, 2006). Furthermore, the fact that assessment processes are largely conducted based on the practitioner's experience and intuitive judgments leads to limited consistency in application and the inability to systematically interpret the data obtained (Andersen, 2009). The use of structured assessment frameworks can contribute to more objective, systematic, and consistent psychological assessment processes by supporting standardized evaluation procedures (Turgut, 1997; Gögebakan and Yildiz, 2025).

Sports psychological assessment is a multi-stage and dynamic process that involves more than just the administration of measurement tools. This process includes data collection, the integration of various psychological dimensions, the creation of individual profiles, and the planning of intervention strategies (Aoyagi et al., 2012). By its very nature, psychological assessment requires a multidimensional and model-based structure (Korkut Owen, 2024). Therefore, the approach needed in sports psychology is not measurement tools based on individual variables, but rather theory-based models that integrate different psychological structures and systematize the assessment process.

Given the inherently multifaceted and layered nature of athlete psychology, the interaction between self-regulatory processes (motivation, concentration, stress and anxiety, anger, mental resilience), social-behavioral functioning (communication, prosocial and antisocial behaviors, sociotropy, autonomy, burnout), and personality/self-structures (self-esteem, self-confidence, perfectionism, narcissism, emotional intelligence) needs to be considered together (Büyükbasmacı, 2023). This approach is theoretically supported by self-regulation theory (Baumeister and Vohs, 2007), trait-situation discrimination (Spielberger, 1972), and psychological wellbeing models (Ryff, 1989). However, studies on how this multifaceted structure can be organized within a structured assessment model that sports psychologists can directly utilize in their practice processes appear to be limited.

At this point, a critical gap emerges in the literature: although robust tools exist for measuring individual psychological variables in sport psychology, structured theory-based assessment models that facilitate the holistic integration of these variables, define a systematic assessment process, and are directly applicable in routine sport psychology practice have not been sufficiently developed (Fletcher and Sarkar, 2012). Sports psychologists may face difficulties in identifying athletes' emotional states (Friesen and Orlick, 2010; Kristiansen and Roberts, 2010). This gap leads sports psychologists to conduct their assessment processes based on individual approaches and poses a significant barrier to standardization in the field (Birrer and Morgan, 2010; Henriksen et al., 2010).

While the model proposed in this study was developed with consideration for the ecological and cultural characteristics of the sports context in Turkey, its theoretical foundations make it adaptable to different cultural and performance contexts. In this regard, the model aims to provide an evaluation framework that draws on the local context yet is applicable on a universal level.

Aiming to address this theoretical and practical gap, this study proposes the SPODAF (Sport Psychological Diagnostic and Assessment Form) model, which enables a multidimensional, layered, and integrative assessment of athletes' psychological state. The proposed model addresses athlete psychology through 16 psychological domains and conceptually organizes them into three overarching categories: self-regulatory processes, social-behavioral functioning, and personality/self-structure. The model offers a structured assessment framework that supports the systematic assessment and interpretation of athletes' psychological state while facilitating the integration of assessment findings into intervention planning.

This study's contribution to the literature can be summarized in three key points. First, it goes beyond the unidimensional measurement approaches commonly used in sports psychology to offer a multidimensional and holistic psychological assessment framework. Second, unlike traditional scales that focus solely on measurement, it proposes a structured assessment process that includes the systematic interpretation of the data obtained and its integration into intervention processes. Third, it presents a practical and operational assessment model that sports psychologists can directly use in routine professional practice.

In this context, the primary objective of the research is to develop a theory-based, multidimensional, and practical model aimed at standardizing psychological assessment processes in sports psychology.

## Method

This study was conducted to develop a theory-based holistic assessment model for structuring the psychological state assessment process in sport psychology and to construct a structured assessment form for practical application based on this model. The study represents the initial conceptual phase of SPODAF (Sport Psychological Diagnostic and Assessment Form) development and adopts a descriptive and constructive research approach grounded in literature synthesis, theoretical framework development, and expert review. Rather than developing and psychometrically validating a measurement scale, the primary objective was to establish a comprehensive assessment model capable of systematically organizing the multidimensional psychological domains considered essential for sport psychologists' assessment and intervention planning.

### Research design

This research was designed as a theory-based model development study. Accordingly, the study does not constitute a scale development or psychometric validation study; therefore, exploratory factor analysis, confirmatory factor analysis, and reliability analyses were intentionally beyond its scope. The primary purpose of the present study was to establish the conceptual structure of the proposed model, develop a structured assessment form based on this framework, and evaluate its theoretical coherence, content relevance, and practical applicability through expert review. Psychometric validation of the proposed model is recommended as a subsequent stage of empirical research.

### Development of the theoretical model

The SPODAF (Sport Psychological Diagnostic and Assessment Form) model was developed through a theory-driven process grounded in the literature on sport psychology, performance psychology, and psychological wellbeing. The study was based on the premise that athletes' psychological state should be assessed through a multidimensional and holistic framework rather than through isolated psychological variables. Accordingly, the conceptual structure of the model was established by integrating psychological domains that have consistently been identified in the sport psychology literature as relevant to athletes' psychological functioning, performance, and wellbeing (Büyükbasmacı, 2023).

The theoretical framework of the model is organized into three overarching categories: self-regulatory processes (motivation, concentration, stress and anxiety, anger, and mental resilience), social-behavioral functioning (communication, prosocial and antisocial behaviors, sociotropy, autonomy, and burnout), and personality/self-structures (self-esteem, self-confidence, perfectionism, narcissism, and emotional intelligence). These categories provide the conceptual foundation for the systematic assessment of athletes' psychological state and facilitate the interpretation of assessment findings for subsequent intervention planning.

### Identification of the 16 psychological domains

The identification of the 16 psychological domains was primarily informed by the conceptual framework presented by Büyükbasmacı (2023), which synthesizes the principal psychological domains addressed in applied sport psychology. Rather than selecting domains through statistical item reduction procedures, the present study adopted a theory-driven approach to preserve the conceptual breadth considered necessary for comprehensive psychological assessment. Consequently, the proposed model incorporates 16 psychological domains representing the multidimensional nature of athletes' psychological state.

The decision to retain all 16 domains was intentional. Reducing or eliminating domains through psychometric procedures at this stage could have compromised the conceptual integrity of the proposed assessment model and limited its capacity to provide sport psychologists with a comprehensive overview of athletes' psychological state. Accordingly, preserving the theoretical structure of the model was prioritized over statistical optimization, while psychometric validation is recommended as a subsequent stage of empirical research.

### Development of the assessment form

Following the establishment of the theoretical model, a structured assessment form was developed to operationalize the proposed framework in applied sport psychology settings. The development process was guided by the objective of creating a practical, standardized, and theory-based assessment form that could be readily integrated into the routine assessment practices of sport psychologists.

Three assessment items were developed for each of the 16 psychological domains, resulting in a total of 48 items. The decision to assign an equal number of items to each psychological domain was intentional and aimed to ensure balanced representation across all domains while facilitating a clear and systematic interpretation of the assessment findings. This structure was designed to enable sport psychologists to evaluate each psychological domain within a consistent framework without overrepresenting or underrepresenting any specific domain.

Item development was guided by the principles of conceptual relevance, behavioral specificity, linguistic clarity, and practical applicability. Accordingly, the items were formulated as short, behavior-oriented statements that could be completed rapidly in applied sport settings while reflecting athletes' current psychological state. In addition, limiting each psychological domain to three items was intended to minimize respondent burden, facilitate rapid administration, and preserve the practicality of the assessment process without compromising the conceptual coverage of the proposed model. The overall structure of the assessment form was therefore designed to support efficient administration under routine field conditions while maintaining the multidimensional nature of the proposed theoretical model.

### Expert review process

To strengthen the theoretical coherence, content relevance, and practical applicability of the proposed assessment model, an expert review process was conducted during the development of SPODAF. Rather than serving as a psychometric content validity study, the expert review was intended to evaluate whether the proposed psychological domains and assessment items were conceptually appropriate and practically applicable within the context of applied sport psychology.

A panel of 12 experts from three different countries participated in the review process. Experts were selected using purposive sampling based on their academic expertise in sport psychology and/or measurement and evaluation. All experts held doctoral degrees and had established records of scholarly publications in their respective fields. Including experts from different countries was intended to obtain feedback from diverse academic perspectives and to improve the conceptual applicability of the proposed model across different cultural contexts.

To facilitate a structured evaluation process, the experts were provided with a table of specifications linking each psychological domain to its corresponding assessment items. Using this framework, the experts evaluated whether the proposed items adequately represented the intended psychological domain. For each item, experts indicated whether it was “Appropriate” or “Not Appropriate” and were invited to provide written comments and recommendations regarding conceptual relevance, linguistic clarity, theoretical consistency, and practical applicability.

### Revision following expert feedback

The experts' recommendations focused primarily on improving conceptual relevance, theoretical representativeness, linguistic clarity, and practical applicability rather than evaluating psychometric properties. Following the review process, revision recommendations were received for nine assessment items. These recommendations mainly involved refining wording, improving conceptual consistency, and enhancing item clarity while preserving the theoretical structure of the proposed model.

All expert comments and recommendations were systematically reviewed by the authors, and revisions were implemented through an iterative refinement process. The final version of the assessment form presented in Appendix 1 reflects the consensus achieved following expert review and revision.

### Structure of the final assessment form

The final SPODAF assessment form consists of 16 psychological domains represented by a total of 48 assessment items, with each psychological domain comprising three behavior-oriented items. This standardized structure enables a balanced assessment across all psychological domains while preserving the multidimensional framework of the proposed model.

The assessment items are presented as short, clear, and behavior-based statements designed to facilitate the rapid evaluation of athletes' current psychological state in applied sport psychology settings. A five-point Likert response format is used to allow athletes to express the intensity of each psychological domain while maintaining ease of administration.

The assessment form was designed to be practical for routine use under field conditions. It can be completed within approximately 3–5 min, allowing sport psychologists to obtain a structured multidimensional psychological profile that can support subsequent assessment, interpretation, and intervention planning. Detailed guidance regarding administration and score interpretation is presented in the “Implementation and Interpretation Framework” section.

### Data analysis

No statistical analyses were conducted as part of this study because the objective was not to develop or psychometrically validate a measurement scale. Instead, the study focused on developing a theory-based assessment model and translating this conceptual framework into a structured assessment form through literature synthesis, theoretical framework development, expert review, and iterative refinement. Consequently, statistical procedures such as exploratory factor analysis, confirmatory factor analysis, reliability analysis, or other psychometric validation techniques were beyond the scope of the present study. Future research may build upon the proposed model by examining its psychometric properties in different athlete populations.

### Ethical considerations

The development of the proposed assessment model involved consultation with academic experts during the expert review process. No data were collected from athletes or other research participants, and no experimental procedures or psychometric data collection were conducted. Expert participation was entirely voluntary, and all experts were informed about the purpose of the study before providing their evaluations.

The comments and recommendations obtained during the expert review process were used solely to refine the conceptual framework and assessment items of the proposed model. Throughout the study, the principles of voluntary participation, confidentiality, anonymity, and responsible scientific conduct were strictly observed.

### Implementation and interpretation framework

#### Intended use

SPODAF is a theory-based assessment form designed to facilitate the multidimensional assessment of athletes' current psychological state. Rather than serving as a diagnostic instrument, the assessment form is intended to provide sport psychologists with a structured overview of multiple psychological domains, thereby supporting systematic assessment and evidence-informed intervention planning. Accordingly, SPODAF functions as a practical decision-support tool for both performance optimization and psychological support processes.

#### Application context

Given the dynamic nature of sport, the assessment form is intended to be administered across different practice contexts, including before or after training sessions, before competitions when appropriate, or during routine consultations conducted by sport psychologists. This flexibility enables the monitoring of athletes' psychological state across different performance environments and time points.

#### Administration procedure

The assessment form is completed individually by athletes under the supervision of a sport psychologist or another appropriately qualified practitioner. Participants respond to each assessment item using a five-point Likert response format that reflects their current psychological state. The assessment generally requires approximately 3–5 min to complete and may be administered in either paper-based or digital formats. Following completion, a multidimensional psychological profile is generated to support subsequent interpretation and intervention planning.

#### Scoring framework

Each psychological domain consists of three assessment items. Domain scores are calculated by summing the responses to the corresponding three items, resulting in scores ranging from 3 to 15 for each psychological domain. The assessment produces a multidimensional psychological profile across all 16 psychological domains rather than a single overall score, enabling sport psychologists to identify the relative strengths and needs of each athlete.

#### Interpretation framework

All assessment items are positively worded; therefore, higher scores indicate higher levels of the corresponding psychological domain. Domain scores may be interpreted according to the following preliminary interpretive framework:

- 13–15 points: Very High
- 10–12 points: High
- 7–9 points: Moderate
- 4–6 points: Low
- 3 points: Very Low

These score ranges are proposed solely as preliminary interpretive guidelines intended to facilitate the practical use of the assessment form during the conceptual stage of model development. They should not be interpreted as empirically validated normative cut-off values or diagnostic thresholds, as the present study does not include psychometric validation. Consequently, future empirical studies should establish evidence-based normative interpretations and score thresholds through comprehensive psychometric analyses conducted across different athlete populations.

Accordingly, the resulting multidimensional psychological profile is intended to assist sport psychologists in identifying relative psychological strengths and psychological domains that may require further attention, thereby supporting individualized assessment, intervention planning, and ongoing psychological monitoring rather than providing definitive diagnostic classifications or clinical decisions.

## Discussion

In this study, the SPODAF model was proposed to make sport psychologists' psychological state assessment processes more systematic, comprehensive, and practical. The model is conceptually organized into three overarching categories—self-regulatory processes, social-behavioral functioning, and personality/self-structures—and provides a multidimensional assessment framework comprising 16 psychological domains. In this regard, the proposed model extends beyond the unidimensional assessment approaches commonly used in sport psychology by integrating multiple psychological domains within a single structured assessment system. Consequently, sport psychologists can obtain a comprehensive overview of an athlete's psychological state through a single assessment. Furthermore, the proposed assessment form is intended to function as a practical guide that sport psychologists can readily incorporate into their routine professional practice, supporting both systematic psychological assessment and intervention planning (Büyükbasmacı, 2023).

The literature indicates that assessment approaches in applied sport psychology primarily focus on individual psychological variables. Constructs such as motivation, anxiety, mental resilience, and self-confidence are typically assessed using separate measurement instruments, making it difficult to obtain a comprehensive understanding of an athlete's psychological state. Moreover, there is a lack of standardized, practice-oriented assessment models that enable sport psychologists to evaluate multiple psychological domains within a single structured framework. The SPODAF model was developed to address this fragmentation by integrating conceptually related psychological domains into a unified assessment model. This approach is consistent with theoretical perspectives emphasizing that psychological assessment should be multidimensional, systematic, and model-based (Korkut Owen, 2024; Demir Başaran, 2025).

One of the key strengths of the proposed model is that it is designed not only to facilitate systematic psychological assessment but also to support intervention planning. By providing a structured overview of athletes' psychological state across multiple psychological domains, the model is intended to help sport psychologists plan and prioritize interventions in a more systematic, consistent, and evidence-informed manner. Andersen (2009) noted that psychological assessment in sport psychology often relies heavily on practitioners' professional experience, which may reduce consistency across assessment practices. In contrast, the structured assessment framework proposed in the present study aims to support more standardized decision-making by providing sport psychologists with rapid, systematic, and practically applicable information. Furthermore, its brief administration time and suitability for routine field settings enhance its practical value in applied sport psychology.

The psychological domains incorporated into the SPODAF theoretical framework are directly related to the fundamental psychological processes that influence athletic performance (Büyükbasmacı, 2023). In particular, self-regulatory processes, including motivation, concentration, stress management, and mental resilience, have consistently been identified as key determinants of athletic performance (Baumeister and Vohs, 2007; Bayraktar and Kurtoglu, 2009; Bozkurt, 2010; Eroglu and Zileli, 2015; Fletcher and Sarkar, 2012; Samur, 2018; Yilmaz and Namli, 2023; Zengin and Kirkbir, 2020). Similarly, social-behavioral domains, such as communication, prosocial behaviors, and sociotropic tendencies, have been associated with team cohesion, perceived social support, and psychological resilience (Akduman et al., 2018; Avcioglu, 2007; Başçi and Geçikli, 2022; Graham, 2011; Gülay and Akman, 2009, p. 1; Katz and McClellan, 1991; Kayabali and Özbey, 2024; Kristiansen and Roberts, 2010; Lynch and Simpson, 2010; McGinnis and Goldstein, 2016). By integrating these interrelated psychological domains within a single structured assessment framework, the proposed model reflects the multidimensional nature of athlete psychology and reinforces the view that sport psychology extends beyond the assessment of isolated performance-related variables.

The SPODAF model is also conceptually aligned with the psychological wellbeing perspective in sport psychology. Ryff, 1989 model of psychological wellbeing emphasizes that individuals should be evaluated not only in terms of performance outcomes but also through multidimensional aspects such as self-acceptance, positive interpersonal relationships, and environmental mastery. Consistent with this perspective, the inclusion of psychological domains such as self-esteem, mental resilience, narcissism, and emotional intelligence within the personality/self-structures category demonstrates that the proposed model adopts a holistic approach to psychological assessment that extends beyond performance and encompasses broader aspects of athletes' psychological functioning.

Seeking expert opinions during the development of the proposed model was an important step in strengthening its theoretical coherence, conceptual relevance, and practical applicability. Expert review is widely recognized as an essential component of both assessment model development and form development, helping to ensure that proposed psychological domains and assessment items adequately represent the intended constructs (DeVellis, 2017; Worthington and Whittaker, 2006; Koçak and Demirdel, 2023). Furthermore, the inclusion of experts from different countries enabled consideration of diverse cultural perspectives and enhanced the potential applicability of the proposed model across different sport contexts.

However, this study has several limitations. First, the proposed model has not yet undergone psychometric validation; therefore, its psychometric properties should be examined in future empirical studies. Furthermore, because the proposed structure represents a theory-based assessment model, its applicability should be evaluated across different sport disciplines, age groups, competitive levels, and cultural contexts. Future research should also investigate its psychometric characteristics through procedures such as confirmatory factor analysis, criterion-related validity, and test–retest reliability. Such studies would strengthen the scientific evidence supporting the proposed model and determine its potential for broader application in both professional sport psychology practice and scientific research.

Future research may also explore the integration of SPODAF into digital assessment platforms, its use in conjunction with real-time psychological monitoring systems, and its adaptation to AI-supported decision-support and assessment processes. As performance analysis systems become increasingly digitalized, integrating structured psychological assessment with data-driven technologies may enhance the efficiency, accessibility, and consistency of psychological assessment in applied sport psychology, while opening new directions for both research and professional practice.

## Conclusion

The present study proposed SPODAF (Sport Psychological Diagnostic and Assessment Form) as a theory-based assessment model designed to structure the psychological state assessment process in applied sport psychology. Rather than functioning as a conventional measurement scale, the proposed model provides a structured assessment framework that conceptually organizes athletes' psychological state across 16 psychological domains grouped within three overarching categories: self-regulatory processes, social-behavioral functioning, and personality/self-structures.

The proposed model addresses an important gap in the sport psychology literature by moving beyond isolated assessments of individual psychological variables toward a multidimensional, systematic, and practice-oriented assessment approach. By integrating multiple psychological domains within a single structured assessment framework, SPODAF is intended to support sport psychologists in obtaining a comprehensive overview of athletes' current psychological state while facilitating systematic assessment, interpretation, and intervention planning. Although the present study represents the conceptual stage of model development and does not include psychometric validation, it provides a theoretical foundation for future empirical research. Subsequent studies should evaluate the psychometric properties of the proposed assessment form across different athlete populations and sport contexts while further examining its practical utility in applied sport psychology. Overall, SPODAF offers a theory-based framework that may contribute to greater consistency, standardization, and multidimensional psychological assessment in both professional practice and future scientific research.

## Recommendations

Future research should focus on evaluating the psychometric properties of the proposed SPODAF assessment form through empirical studies involving different athlete populations. Procedures such as exploratory and confirmatory factor analyses, reliability assessment, criterion-related validity, construct validity, and test–retest reliability should be conducted to determine the measurement properties of the assessment form while preserving the conceptual structure of the proposed theoretical model.

The applicability of SPODAF should also be examined across different sport disciplines, competitive levels, age groups, and cultural contexts to evaluate its generalizability and practical utility. Comparative studies involving individual and team sports, amateur and elite athletes, as well as athletes from different cultural backgrounds, may further strengthen the empirical evidence supporting the proposed model.

Future developments may include adapting SPODAF to digital assessment platforms, mobile applications, and web-based systems to facilitate rapid administration, automated profile generation, and longitudinal monitoring of athletes' psychological state. Integration with data-driven performance monitoring systems and AI-supported decision-support technologies may further enhance the efficiency and practical applicability of structured psychological assessment in applied sport psychology.

Finally, future studies should investigate the integration of SPODAF with complementary sources of information, including coach observations, performance indicators, and relevant physiological or biological measures, to support a more comprehensive and multidimensional understanding of athletes' psychological functioning. The applicability of the proposed model should also be examined among diverse populations, including youth athletes, athletes with disabilities, recreational participants, and other performance populations beyond elite sport.

## Data Availability

The original contributions presented in the study are included in the article/Supplementary Material, further inquiries can be directed to the corresponding author.
