# Supplementary material for: Structuring the psychological “State” assessment process for sports psychologists: a proposal for a theory-based holistic model
Source: Front Psychol. 2026 Sep 21;17:1919803. doi: 10.3389/fpsyg.2026.1919803 (PMC13634812; doi:10.3389/fpsyg.2026.1919803)
Supplement: Supplementary file 1 [file Supplementary_File_1.pdf]

Appendix-1 SPODAF (Sport Psychological Diagnostic and Assessment Form)

Questions						
**1. Motivation Sub-Dimension**		1	2	3	4	5
1	Even when I'm feeling at my lowest, I can motivate myself like a psychologist by doing mental exercises.					
2	The setbacks I face never affect my motivation.					
3	I hope to encounter challenges along the way to my goal; they make me stronger.					
**2. Concentration Sub-Dimension**						
4	Even if my efforts yield negative results, I don't lose my concentration.					
5	Even though I might consider other goals more appropriate, I can't let that distract me from my current focus.					
6	Even if I receive news of a disaster, I remain unfazed and focus on my goals.					
**3. Anger Sub-Dimension**						
7	Even though all the circumstances are challenging, I'm not getting angry.					
8	I can maintain self-control when I'm angry.					
9	Even frustrating events and situations shouldn't make people angry.					
**4. Stress Sub-Dimension**						
10	I don't get stressed out, even during the most important moments of my life.					
11	I recognize when I'm about to feel stressed and take steps to cope.					
12	Everything is going well in my life.					
**5. Anxiety Sub-Dimension**						
13	Life isn't a bad place; all the other people have my best interests at heart.					
14	I don't feel anxious even when my performance is poor.					
15	Even if I were bullied, it wouldn't affect me. I'm sure no one would hurt me.					
**6. Prosocial and Antisocial Sub-Dimension**						
16	I support even the people who have wronged me.					
17	I encourage people to work together.					
18	If someone is being treated unfairly, I will definitely step in—even if it doesn't concern me personally or goes against my interests.					
**7. Burnout Sub-Dimension**						
19	I really love working out; I have endless energy.					
20	If I see someone suffering from burnout, I can't just ignore them—I'll have a heart-to-heart talk with them.					
21	Life isn't a difficult place, in my opinion.					
**8. Contact Sub-Dimension**						
22	Even if I'm feeling down, I can still communicate positively with the people responsible for it.					
23	I often use empathetic behavior throughout the day.					
24	Even if the person in front of me treats me very badly, I must find a way to win them over.					
**9. Mental Resilience Sub-Dimension**						
25	Even though adversity gets me down, I can still stay mentally resilient.					
26	Whenever I feel burned out, a voice inside me warns me that I need to keep fighting.					
27	I enjoy being my own doctor throughout the day.					
**10. Perfectionism Sub-Dimension**						
28	I never have unrealistically high expectations in any situation.					
29	I don't push myself too hard just to make my work perfect.					
30	When I fail, I don't judge myself harshly.					
**11. Sociotropic Personality Sub-Dimension**						
31	Even when I'm at my most irritable, I'm still able to interact positively with those around me.					
32	I have no qualms about being alone.					

33	I don’t really care much about whether people approve of me.					
**12. Autonomous Personality Sub-Dimension**						
34	People who often ask for help while handling their own affairs are on the wrong track.					
35	Even if it means disrupting my work, I will stand up for everyone’s rights.					
36	I am deeply saddened when I see people who are dependent on others in their behavior.					
**13. Self-esteem Sub-Dimension**						
37	If I lose my self-respect, I’ll end up in a pitiful state.					
38	Self-respect is undoubtedly the most important emotion.					
39	I consider whether all the actions I've taken are appropriate for me.					
**14. Narcissism Sub-Dimension**						
40	I don’t consider myself the most talented among the athletes.					
41	I can't say I like myself in every aspect.					
42	I don’t criticize athletes who fail.					
**15. Self-confidence Sub-Dimension**						
43	I am a very confident person.					
44	I am fully confident in my ability to succeed, but I do fail sometimes.					
45	The situations I encounter do not undermine my self-confidence.					
**16. Emotional Intelligence Sub-Dimension**						
46	I spend time trying to understand and sense other people’s feelings.					
47	I have never been affected by the events I experienced in the past.					
48	I regularly take the time to identify my weaknesses.					
